# Family dynamics in a multi-ethnic Asian society: comparison of elderly CKD patients and their family caregivers experience with medical decision making for managing end stage kidney disease

**DOI:** 10.1186/s12882-019-1259-4

**Published:** 2019-03-01

**Authors:** Semra Ozdemir, Tazeen H. Jafar, Lina Hui Lin Choong, Eric Andrew Finkelstein

**Affiliations:** 1 Lien Centre for Palliative Care, Duke-NUS Medical School, 8 College Road, Singapore, 169857 Singapore; 2 Programme in Health Services and Systems Research Programme, Duke-NUS Medical School Singapore, 8 College Road, Singapore, 169857 Singapore; 3 Department of Renal Medicine, Singapore General Hospital, 20 College Road, Singapore, 169856 Singapore

**Keywords:** End stage kidney disease, Elderly, Family caregivers, Decision making, Treatment preferences

## Abstract

**Background:**

Elderly end stage kidney patients face a decision concerning whether or not to initiate dialysis. In Asia, this decision is highly influenced by family caregivers. The objective of this paper was to understand patients’ experience with and preferences for family involvement in treatment decisions, and via a series of hypothetical vignettes, to identify whether there was discordance in treatment preferences between patients and their caregivers, and how any potential conflicts were reconciled.

**Methods:**

We conducted a survey with 151 elderly (aged ≥65) chronic kidney disease patients and their caregivers at outpatient renal clinics. The survey asked, when making treatment decisions, whom they wish makes the final decisions (i.e., preference) and who usually makes the final decisions (i.e., experience). The survey also presented a series of choice vignettes for managing patient’s condition and asked respondents to choose between two hypothetical treatment profiles in each vignette. Patients and caregivers were first interviewed separately in tandem, and then were brought together to choose a treatment jointly for vignettes where the initial treatment choice differed within the dyad. We used multivariate regressions to investigate the predictors of discordance and reconciliation.

**Results:**

We found that most (51%) patients preferred and experienced (64%) significant involvement from caregivers. However, 38% of patients preferred to make final decisions alone but only 27% of patients did. In the hypothetical vignettes, caregivers chose the more intensive option (i.e., dialysis) more than patients did (26% vs 19%; *p* < 0.01). Overall, 44% of the dyads had discordance in at least 3 vignettes, and the odds of discordance within patient-caregiver dyads was higher when caregivers chose dialysis or treatment with the higher cost (*p* < 0.01). In half the cases, discordance resolved in the patients’ favor, and this was more likely to be the case if the patient was employed and wanted to be in charge of final decisions (*p* < 0.01).

**Conclusions:**

Our results highlight the important role of caregivers in decision-making but also the potential for them to overstep. Clinicians should be aware of this challenge and identify strategies that minimize the chances that patients may receive treatments not consistent with their preferences.

**Electronic supplementary material:**

The online version of this article (10.1186/s12882-019-1259-4) contains supplementary material, which is available to authorized users.

## Background

Dialysis is a life-saving treatment option and is considered first-line of treatment for most end stage kidney disease (ESKD) patients [1]. However, several studies show little or no survival benefits of dialysis for elderly patients (≥75) with multiple comorbidities when compared to conservative management (CM), which focuses on symptom management, dietary control and supportive care [2–5]. Compared to CM, dialysis patients also experience higher rates of hospitalization [6] and higher costs [7, 8]. For these reasons, international bodies, such as U.S. Renal Physicians Association recognize CM as a viable treatment alternative for elderly ESKD patients with multiple comorbidities [9].

For many patients, the decision of whether or not to initiate dialysis is based on more than just clinical and economic factors [10]. Family caregivers often play an active role in helping synthesize complex information and make treatment decisions [11, 12]. This is especially the case for non-English speaking patients, older patients and patients with lower education [13–15]. Recognizing the important role of families, the National Kidney Foundation and other relevant bodies recommend family involvement in counselling sessions on ESKD management [16]. Whereas this again sounds like a reasonable approach, it raises concerns over patient autonomy and how treatment decisions are reconciled when patients and caregivers disagree. Evidence from oncology literature suggests that in many Asian countries, including Singapore, the setting of this study, patient autonomy is often not absolute and, in many cases, caregivers and physicians jointly make treatment decisions in the absence of the patient [15, 17–20]. Although caregivers may believe they are acting in the best interests of the patient this may not be the case [21]. Studies from different therapeutic areas have shown that caregivers have different priorities than patients and tend to favor more aggressive treatments over conservative options, perhaps to avoid regret for not pursuing all available options [22–24]. For example, one study conducted with patients in Canada showed that patients who experienced regret with dialysis also reported that treatment was based on the family’s recommendation [11]. Furthermore, evidence shows that caregivers are more willing than patients to use family resources for treatment expenses. In a study conducted with advanced cancer patients in Singapore, Malhotra et al. (2018) [25] found that caregivers had greater willingness to pay than patients to extend patient’s life and to improve their quality of life. However, there is limited evidence on kidney patients’ experiences with and preferences for family involvement in decision making in general and specific to the decision to opt for dialysis or CM.

To address this gap, we administered a survey to elderly patients with chronic kidney disease (CKD) and their family caregivers. We first asked patients and caregivers 1) their preferences for and 2) experience with health-care decision making to test the hypothesis that there would be discordance between preferences and experience with decision making Specifically, we hypothesize that (H1) patients prefer greater involvement in making final decisions than what their caregivers want for them, and, as a result (H2) patients prefer greater involvement in final decisions than they currently experience.

We then asked both patients and caregivers to choose between hypothetical treatment profiles of dialysis and CM in a series of choice vignettes. We separately asked patients and their caregivers to choose their preferred treatment profile in each vignette. We then brought them back and asked them to discuss those vignettes where the preferred treatment profile differed and to make a joint choice in efforts to determine whose preferences tend to dominate treatment decisions. Based on the vignettes, we tested the following hypotheses: (H3) Caregivers will choose the more aggressive option (a form of dialysis) over CM more than patients will; and (H4) the odds of discordance within patient-caregiver dyads will be higher when caregivers choose dialysis (i.e. caregiver would push for more aggressive treatment) or the treatment with the higher cost (i.e. patients would not want to be a financial burden to the family). We further hypothesize that due to power dynamics (H5) discordance is less likely to be reconciled in patient’s favor if the patient is older, sicker, or has no formal education (i.e., has less power), and is more likely to be reconciled in patient’s favor if the patient is male, employed or wants to be in charge of final decision making (i.e., has more power) [26].

If findings show that patient autonomy is being compromised and/or there is a high degree of discordance, then greater efforts should be made to ensure that patients are appropriately included in the decision-making process.

## Methods

### Participants and setting

A survey was administered via face-to-face interviews between May and November 2015 at the Department of Renal Medicine outpatient clinics at the Singapore General Hospital, the largest tertiary hospital of Singapore. Patients aged 65 or older who were diagnosed with Stage 3B to 5 CKD (eGFR < 45 ml/min/1,73 m2) and not receiving renal replacement therapy were identified from their medical records and were approached by trained interviewers. Patients were further screened for being aware of prognosis, never having been on dialysis, and being cognitively intact which was tested via the Abbreviated Mental Test [27]. Inclusion criteria for caregivers included being aged 21 years and over and being primarily involved in providing care, ensuring provision of care to the patient, or making decisions regarding patient’s treatment/care. The survey was administered in English, Mandarin and Malay to 161 patient–caregiver dyads. The survey was reviewed and approved by SingHealth’s Institutional Review Board.

### Survey development

After a brief introduction of ESKD and alternative treatments for managing ESKD, patients and caregivers were asked, when making decisions about health care (of the patient), whom they wish makes the final decisions (i.e., preference for decision making) and who usually makes the final decisions (i.e., experience with decision making). Response options were “me (patient) only”, “my (patient’s) family only”, “my (the) doctor only”, “me (patient) and family only”, “me (patient) and doctor only”, “my (patient’s) family and doctor only”, and “me (patient), family and doctor”.

**Fig. 1:**
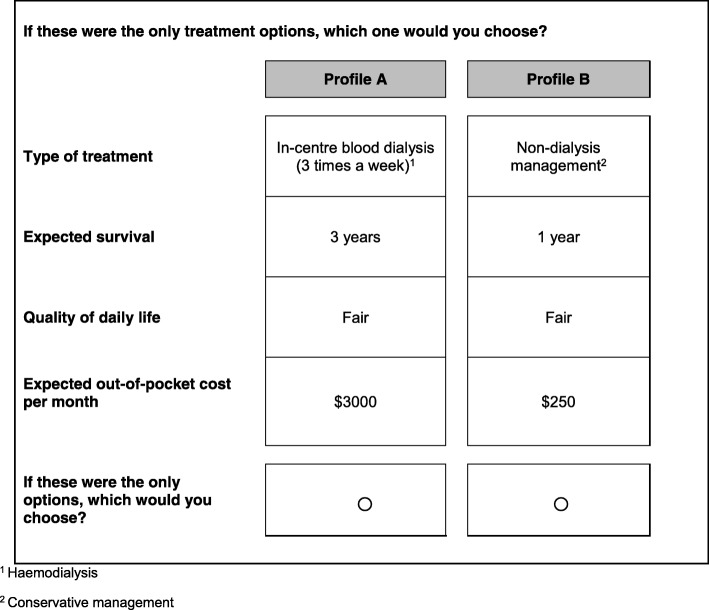
Sample Treatment-Choice Vignette

The survey asked respondents to assume that they (the patient) have kidney failure and presented a series of choice vignettes for managing their condition. Each vignette asked respondents to choose between two hypothetical ESKD treatment profiles (See Fig. 1 for an example choice vignette). Each profile was defined by four attributes: 1) type of treatment (hemodialysis, peritoneal dialysis or CM), 2) quality of daily life (poor, fair, good or very good), 3) expected survival (1, 3, 6 or 10 years for dialysis and 1 or 3 years for CM), and 4) expected out-of-pocket medical cost per month. Quality of daily life ranged from “poor”, when a patient is not able to do activities that are important to them, to “very good”, when a patient is able to do all of the activities that are important to them. For expected survival, dialysis alternatives offered longer survival than CM as might be the case for younger patients or patients with low comorbidity. The cost levels ranged from $250 to $2000 per month for CM and from $700 to $7000 per month for dialysis consistent with the higher costs associated with dialysis (Table 1).

**Table 1:** Treatment Attributes and Levels

Attributes	Levels
Type of treatment	• In-centre blood dialysis (i.e. Haemodialysis)^a^ • Water-bag dialysis at home (i.e. Peritoneal dialysis) • Non-dialysis management (i.e. Conservative management)
Expected survival^b^	• Dialysis: 1 year, 3 years, 6 years, 10 years • Conservative management: 1 year, 3 years
Quality of daily life	• Poor • Fair • Good • Very good
Expected out-of-pocket cost per month^c^	• Dialysis: S$700, S$1500, S$3000, S$7000 • Conservative management: S$250, S$500, S$1000, S$2000

^a^The types of treatment were labelled such that it will be easier for respondents to understand and remember during the survey

^b^The levels of the expected survival differed between dialysis and conservative management

^c^The levels of the out-of-pocket cost differed between dialysis and conservative management

Patients and caregivers were first surveyed separately by two interviewers in tandem. After the completion of the individual interviews, patient-caregiver dyads were brought together and they were asked to choose an option jointly for the choice vignettes that they provided different answers. They were also given the option to choose “no consensus” in the joint interviews. This option was added after encounters with dyads who could not agree on an option after several minutes of discussion during the cognitive interviews.

Survey was developed based on consultations with three nephrologists, a social worker, and a nurse counsellor who work with ESKD patients, and findings from cognitive interviews. Cognitive interviews were conducted with 10 patients and caregivers and were used to identify the attribute levels and to test the length and wording of the survey instrument. The questionnaire was first developed in English and then was translated into Mandarin and Malay by professional translators and were further reviewed by team members who were native speakers in these languages.

The hypothetical choice vignettes were constructed via an experimental design that was created using SAS [28]. Each respondent was randomized into one of the 8 sets of 9 choice vignettes to reduce the cognitive burden. Two of the choice vignettes were used to test whether respondents paid attention to the choice scenarios [29, 30]. This test requires that if patients choose Profile A to Profile B in a choice vignette, and choose Profile B to Profile C in another choice vignette, then they should also prefer Profile A to Profile C in a third choice vignette. Those who fail the attention test were dropped from the sample. Lastly, we asked questions on comorbidities using a simplified version of the conditions included in the Charlson Comorbidity Index, socio-economic characteristics and how caregivers are related to their patients (Additional file 1).

### Analysis

For hypotheses H1 and H2, patient involvement was defined in two ways: 1) Patient makes final decisions alone (i.e., “patient only”); and 2) patient involves in final decisions in any capacity (e.g., alone or with their family and/or physician). We then compared the percentages were using a McNemar’s test, which is the appropriate version of the chi-squared test in the presence of matched pairs.

When analyzing the vignettes, we first presented the number and percentage of dyads with 1 to (maximum possible) 9 discordant treatment choices. We then tested H3 using a test of proportions to investigate whether caregivers chose dialysis over CM more than patients did.

We used a binomial logistic regression to test H4 using all participant responses. The dependent variable was 1 if a patient and his/her caregiver provided a different treatment option in a choice vignette (i.e. discordance), 0 otherwise. The independent variables of interest were two dummy variables; one indicating a caregiver choosing dialysis over CM and another indicating a caregiver choosing the treatment with the higher cost over the treatment with the lower cost in a choice vignette. The model controlled for other observable patient and caregiver characteristics and differences in expected survival and quality of daily life between the two treatment profiles in a choice vignette. Standard errors were adjusted for having multiple observations for each dyad.

To quantify the degree of reconciliation, we compared the joint choices of a patient-caregiver dyad to patient (alone) and caregiver (alone) choices. To test H5 we used a negative binomial regression model where the dependent variable was the number of times discordance reconciled in the patient’s favor. If discordance was reconciled in caregiver’s favor or no consensus was reached, then it was recorded as 0. This analysis was limited to dyads who had at least one vignette with discordance. Independent variables included dummy variables representing older age (80 and above), lower health status, having no formal education, being employed (i.e. having a full-time or part-time job or being self-employed), being male and wanting to be in charge of final decision making. Lower health status was defined as being in the 25th percentile of the sample quality-adjusted life-years (QALY) which was measured using EQ-5D-3L questions and value sets estimated in Luo et al. [31]. Patients who reported that they alone would like to make the final decisions about health and health care were considered as those who want to be in charge of final decision making. The model controlled for the number of opportunities each dyad had to reach consensus via the number of discordance within a dyad.

All analyses were conducted at STATA 14. Statistical significance was measured at the 5% level.

## Results

### Patient and caregiver demographics

Table 2 presents the patient and caregiver characteristics for 151 dyads, after removing 4 patients and 6 caregivers who failed the attention test. Most patients (62%) were male, 20% were aged 80 and above, 16% had no formal education and only 10% reported having a full-time or part-time job or being self-employed. Most patients (82%) reported at least one comorbidity (in addition to CKD). The mean QALY weight (0.82) was within the range found in the literature [32]. Majority of the caregivers were either spouses (40%) or children (53%) of the patients.

**Table 2:** Patient and Caregiver Characteristics

	Patients	Caregivers
CKD stages based on eGFR		
Stage 3B	29%	*NA*
Stage 4	45%	*NA*
Stage 5	26%	*NA*
Comorbidities		
No comorbidities	18%	*NA*
1 comorbidity	38%	*NA*
2 or more comorbidities	44%	*NA*
Diabetes	64%	*NA*
Cerebrovascular disease	11%	*NA*
Heart failure	13%	*NA*
Coronary artery disease	21%	*NA*
Dysrhythmia	14%	*NA*
Other cardiac conditions	3%	*NA*
Chronic obstructive pulmonary disease	7%	*NA*
Liver disease	7%	*NA*
Gastrointestinal bleeding	6%	*NA*
Peripheral vascular disease	12%	*NA*
Cancer	7%	*NA*
Age, mean (SD)	74 (6)	56 (13)
Aged 80 and above	20%	2%
QALY, mean (SD)	0.82 (0.20)	*NA*
25th percentile QALY	0.69	*NA*
Male	62%	19%
Education		
No formal education	16%	5%
Primary and secondary education	72%	47%
Above secondary education	12%	48%
Employment		
Full-time, part-time, self-employed	10%	55%
Other (homemaker, retired, unemployed)	90%	45%
Relationship to Patient		
Spouse	*NA*	40%
Child	*NA*	53%
Sibling	*NA*	2%
Other	*NA*	5%

### Results on decision-making

Table 3 presents the descriptive statistics on decision-making. Results reveal that 38% of patients preferred making final decisions alone, 36% (CI 29–45) preferred making final decisions together with their family and/or physicians, and 26% (CI 20–34) preferred leaving final decisions entirely to their family and/or physician. Most patients (64%) (CI 55–71) and their caregivers (73%) (CI 65–80) reported family involvement in making final decisions in some capacity.

**Table 3:** Patient and Caregiver Experience of and Preferences for Decision Making

	Patients	Caregivers
When making decisions about your health and health care, *who do you want to make the final decisions*? (i.e., preference for decision making)		
Patient only	38% (31–47)^a^	40% (32–48)
Patient’s family only	17% (12–24)	13% (8–20)
The doctor only	8% (4–13)	3% (1–7)
Patient and family only	13% (8–19)	17% (12–24)
Patient and doctor only	3% (1–7)	3% (1–7)
Patient’s family and doctor only	1% (0.2–5)	6% (3–11)
Patient, family and doctor	20% (14–27)	18% (13–26)
When making decisions about your health and health care, *who usually makes the final decisions*? (i.e., experience with decision making)		
Patient only	27% (20–34)	21% (15–29)
Patient’s family only	5% (2–10)	8% (4–13)
The doctor only	5% (2–10)	1% (0.02–4)
Patient and family only	16% (10–23)	19% (13–26)
Patient and doctor only	5% (2–9)	5% (2–10)
Patient’s family and doctor only	6% (3–11)	7% (3–12)
Patient, family and doctor	36% (29–45)	39% (31–47)

^a^95% confidence intervals are presented in parentheses

The McNemar’s chi-squared test results reject H1 that patients prefer greater involvement in making final decisions than what caregivers want for them. This is rejected for “patient only” involvement (38% (CI 31–47) patients vs 40% (CI 32–48) caregivers; *p* value = 0.40) and for patient involvement in any capacity (74% (CI 66–80) patients vs 78% (CI 71–84) caregivers; *p* value = 0.91).

Tests of hypothesis (H2) that patients prefer greater involvement in making final decisions than they currently experience generated mixed results. Fewer patients prefer to be involved in final decisions in any capacity (74% (CI 66–80) prefer vs 83% (CI 77–89) experience; *p* value < 0.01). However, among those who would like to be involved, more patients prefer to make final decisions alone than those who currently do (38% (CI 31–47) prefer alone vs 27% (CI 20–34) experience alone; *p* value = 0.01). In general, patients prefer a more unilateral (i.e., patient only, family only, or doctor only) decision-making rubric than a collective one (i.e., more than one person/unit). While 64% (CI 55–71) of the patients prefer a unilateral decision making rubric only 37% (CI 29–45) reported having one (*p* value < 0.01).

### Results on treatment preferences

Table 4 shows that 81% (CI 74–87) of the dyads had at least one discordant treatment choice out of 9 possible cases and 44% (CI 36–53) of the dyads had discordance in at least 3 vignettes. Although no dyad had more than 7 discordant choices, the results suggest that discordance within dyads is fairly common. Investigating treatment choices more closely shows that caregivers chose dialysis over CM significantly more than patients did (26% (CI 23–28) for caregivers vs 19% (CI 16–21) for patients; *p* value < 0.01), confirming H3. Consistent with H4, the binomial logit estimates show that the odds of dyads having discordance were higher if caregivers chose a form of dialysis over CM (*p* value < 0.01) or chose the treatment with the higher cost CM (*p* value < 0.01) (Table 5). No other variables, except the difference in quality of daily life between the treatments in a choice vignette, were statistically significant.

**Table 4:** Number of Discordance at the Dyad Level

Number of Discordance Level	Percent
None	18% (13–26)^a^
1	25% (18–33)
2	12% (7–18)
3	17% (11–23)
4	11% (7–17)
5	7% (4–13)
6	7% (3–12)
7	3% (1–7)
Total	100%

^a^95% confidence intervals are presented in parentheses

**Table 5:** Binomial Logit Model Estimates on Odds of Discordance between Patients and Caregivers

	Odds ratio	Standard Error	*P* Value
Caregiver chose dialysis over CM	4.303	1.017	0.000
Caregiver chose the treatment with the higher cost	4.861	0.951	0.000
Aged 80 and over	0.957	0.199	0.832
Lower health status (in the 25th QALY percentile)	1.330	0.278	0.173
No formal education	0.814	0.188	0.372
Full-time, part-time or self employed	0.891	0.232	0.658
Male	1.260	0.265	0.271
Difference in expected survival between treatment options	1.027	0.029	0.334
Difference in quality of daily life between treatment options	1.222	0.086	0.004
Constant	0.079	0.019	0.000
Log pseudolikelihood	− 600		
Pseudo R-squared	0.235		

Results further showed that discordance resolved in patient’s favor 50% (CI 45–56) of the time, in caregiver’s favor 32% (CI 27–36) of the time and no consensus was reached 18% (CI 14–23) of the time. The only significant predictors of reconciliation were employment status and wanting to be in charge of final decisions (H5). Discordance was more likely to resolve in the patient’s favor if the patient was employed (*p* value = 0.04), presumably as this decreased the likelihood that the patient was financially dependent, and if the patient wanted to be in charge of final decision making (*p* value < 0.01) (Table 6).

**Table 6:** Negative Binomial Model Estimates on The Number of Times Discordance Resolving in Patient’s Favor

	Odds ratio	Standard Error	*P* Value
Aged 80 and over	0.349	0.239	0.143
Lower health status (in the 25th QALY percentile)	−0.125	0.222	0.572
No formal education	−0.441	0.321	0.170
Full-time, part-time or self employed	0.642	0.313	0.040
Male	−0.211	0.214	0.325
Patient wants to be in charge (i.e. the only decision maker)	0.410	0.198	0.038
Number of discordance within a dyad	0.387	0.056	0.000
Constant	−1.094	0.294	0.000
Log likelihood	− 178		
Pseudo R-squared	0.134		

## Discussion

The primary goal of this paper was to compare patients’ and family caregivers’ preferences for and experience with decision making in efforts to understand whether CKD patient preferences for decision making are respected and whether the family may be overstepping their role. Using a series of hypothetical vignettes, we compared decisions of patients and their caregivers for dialysis or CM to quantify discordance in treatment preferences, and how discordance is likely to be reconciled should it occur when real decisions are being made.

Our findings suggest that elderly CKD patients in Singapore do not feel that they are shut out from health care discussions. The majority of participants reported preferring and experiencing significant involvement from caregivers. Although this may contradict the Western perspective of patient autonomy, in Asian societies where filial piety dictates taking care of one’s parents and family, elderly patients may regard decision-making as the caregivers’ duty and may expect caregivers to make treatment decisions on their behalf. We should note, however, that among patients who would like to be involved in decision making, about half preferred to be the only one responsible for making final decisions, and this preference was not always met. Evidence shows that, even in Western societies, ESKD patients are not involved in treatment decisions in a capacity they prefer [14, 33]. Patients reported that the decision belonged to the physician or they were ‘convinced’ to initiate dialysis by physicians [13, 33, 34]. Providers should make every effort to identify patients who would like to be part of the decision making process and make sure that their preference are respected.

Despite the finding that patients were generally happy with caregivers making final decisions, our findings cast doubt that this approach will optimize patient welfare. We find that the frequency of discordance in treatment choices was fairly common within patient-caregiver dyads. Our results show that caregivers tend to choose dialysis more than patients do confirming that caregivers tend to push for aggressive treatments. The results also confirm that patients did not want to be a financial burden to their families and higher treatment costs were more likely to lead to discordance within the family. Patients and families should be encouraged to have discussions with social workers on the treatment costs and how different treatment options may affect household finances, ideally, before making a treatment decision so that all household members have similar expectations.

Our findings also show that, in half the cases, the patient was able to talk the caregiver out of an initial treatment choice. This is an ideal outcome in the presence of shared decision-making, but suggests that if the caregiver alone is left to make the final treatment choice, the patient may undergo a treatment that is not the preferred option and may experience decision regret as found in the study cited in the introduction [11]. Results further suggest that this outcome may be more likely when the patient is not financially independent, as that may put the patient at a disadvantage when negotiating with the caregiver.

What then is the ideal strategy for counselling elderly ESKD patients and their caregivers in Asian cultures? First, it is important to recognize that dialysis is different from many medical procedures in that it requires a commitment from the family in order to ensure optimal outcomes. Families should be included in the decision making process, especially given that the choice of treatment is non-obvious for many elderly comorbid patients, and treatment places a burden on caregivers as well. Second, it is important to recognize that the Asian context differs from the West and that patient autonomy is not absolute. Therefore, we suggest that the ideal strategy for helping patients and families choose ESKD treatment pathways may be to follow the approach used in our study: 1) educate both patients and caregivers about the available options, including all relevant benefits, risks, and costs, 2) discern patient’s preferred role in decision-making and convey that information to the family, 3) independently elicit treatment preferences from both patient and family, and 4) work towards a joint decision in cases of misalignment while adhering to the patient’s preferred role in decision-making. This approach aims to minimize chances of regret when treatment choices are non-obvious.

This study has several limitations. First, the vignette questions were hypothetical and respondents may not have taken the tradeoffs as seriously as they would had they been real treatment choices. Whereas this is a possibility, the fact that 18% of the discordance was not reconciled among the group shows that patients and caregivers were expressing strong views even for these hypothetical scenarios. A second limitation is that we used a convenience sample of CKD patients seen at the largest hospital in Singapore. We chose CKD patients who had not yet progressed to ESKD largely as a means to facilitate data collection in a timely manner. Future studies could attempt to uncover discordance using more representative samples of ESKD patients/caregivers faced with real treatment choices. This would be an important extension of our work given that our study suggests a strong preference, even among caregivers, for CM, whereas in Singapore most elderly ESKD patients undergo dialysis. We suspect this dichotomy results because our study offered participants very clear choices where relevant outcomes were known with certainty, whereas patients/caregivers making choices in the real world are likely to be doing so with far more uncertainty in all relevant outcomes and after receiving input from clinicians, who often promote dialysis over CM given it’s clear survival advantage among younger, healthier patients [3]. Lastly, the findings may not generalize to other settings with different familial structures and health-care systems. However, it is likely that patients who are employed and hence presumably have lower financial dependence, and who would like to be in charge of final decisions would be less likely to be swayed easily by others, including their family members, in any culture.

## Conclusion

Our study results clearly highlight the important role of caregivers in facilitating optimal treatment choices for elderly ESKD patients but also the potential for them to overstep. Clinicians should be aware of this challenge and identify strategies that minimize the chances of treatment regret among elderly ESKD patients. Future studies should explore strategies on how best to include family caregivers in decision making while protecting patient autonomy in Asian societies.

## Additional file

Additional file 1: Patient Survey Instrument. The survey instrument administered to the patient participants in this study. (PDF 273 kb)

## Abbreviations

CI: Confidence interval
CKD: Chronic kidney disease
CM: Conservative management
eGFR: Estimated glomerular filtration rate
ESKD: End stage kidney disease
QALY: Quality adjusted life years

## References

[1] Canaud B, Tong L, Tentori F (2011). Clinical practices and outcomes in elderly hemodialysis patients: results from the Dialysis outcomes and practice patterns study (DOPPS). Clin J Am Soc Nephrol.

[2] Murtagh FE, Marsh JE, Donohoe P (2007). Dialysis or not? A comparative survival study of patients over 75 years with chronic kidney disease stage 5. Nephrol Dial Transplant.

[3] Chandna SM, Da Silva-Gane M, Marshall C, et al. Survival of elderly patients with stage 5 CKD: comparison of conservative management and renal replacement therapy. Nephrol Dial Transplant. 2010; gfq630. 10.1093/ndt/gfq630 PMC3084441 21098012

[4] Shih C-J, Chen Y-T, Ou S-M (2014). The impact of dialysis therapy on older patients with advanced chronic kidney disease: a nationwide population-based study. BMC Med.

[5] Hussain JA, Mooney A, Russon L (2013). Comparison of survival analysis and palliative care involvement in patients aged over 70 years choosing conservative management or renal replacement therapy in advanced chronic kidney disease. Palliat Med.

[6] Carson RC, Juszczak M, Davenport A (2009). Is maximum conservative management an equivalent treatment option to dialysis for elderly patients with significant comorbid disease?. Clin J Am Soc Nephrol.

[7] Jha V, Garcia-Garcia G, Iseki K (2013). Chronic kidney disease: global dimension and perspectives. Lancet.

[8] Brunori G, Viola BF, Maiorca P (2008). How to manage elderly patients with chronic renal failure: conservative management versus dialysis. Blood Purif.

[9] Renal Physicians Association. Shared decision-making in the appropriate initiation of and withdrawal from dialysis. Clinical practice guideline. Second edition. Maryland; 2010.

[10] Tonkin-Crine S, Okamoto I, Leydon GM (2015). Understanding by older patients of dialysis and conservative management for chronic kidney failure. Am J Kidney Dis.

[11] Davison SN. End-of-life care preferences and needs: perceptions of patients with chronic kidney disease. Clin J Am Soc Nephrol. 2010; CJN. 05960809. 10.2215/CJN.05960809 PMC2827591 20089488

[12] Morton RL, Tong A, Webster AC (2011). Characteristics of dialysis important to patients and family caregivers: a mixed methods approach. Nephrol Dial Transplant.

[13] Song M-K, Lin F-C, Gilet CA (2013). Patient perspectives on informed decision-making surrounding dialysis initiation. Nephrol Dial Transplant.

[14] Orsino A, Cameron JI, Seidl M (2003). Medical decision-making and information needs in end-stage renal disease patients. Gen Hosp Psychiatry.

[15] Chong JA, Quah YL, Yang GM, et al. Patient and family involvement in decision making for management of cancer patients at a centre in Singapore. BMJ Support Palliat Care. 2013; bmjspcare-2012-000323. 10.1136/bmjspcare-2012-000323 24644164

[16] Gilmore J (2006). KDOQI clinical practice guidelines and clinical practice recommendations-2006 updates. Nephrol Nurs J.

[17] Back MF, Huak CY (2005). Family centred decision making and non-disclosure of diagnosis in a south east Asian oncology practice. Psychooncology.

[18] Chiu TY, Hu WY, Cheng SY (2000). Ethical dilemmas in palliative care: a study in Taiwan. J Med Ethics.

[19] Hu W-y, T-y C, R-b C (2002). Solving family-related barriers terminal Cancer in Taiwan. Cancer Nurs.

[20] Pang MC (1999). Protective truthfulness: the Chinese way of safeguarding patients in informed treatment decisions. J Med Ethics.

[21] Miura Y, Asai A, Matsushima M (2006). Families' and physicians' predictions of dialysis patients' preferences regarding life-sustaining treatments in Japan. Am J Kidney Dis.

[22] Yun YH, You CH, Lee JS (2006). Understanding disparities in aggressive care preferences between patients with terminal illness and their family members. J Pain Symptom Manag.

[23] Tang ST, Liu T-W, Lai M-S (2005). Concordance of preferences for end-of-life care between terminally ill cancer patients and their family caregivers in Taiwan. J Pain Symptom Manag.

[24] Fried TR, Bradley EH, Towle VR (2003). Valuing the outcomes of treatment: do patients and their caregivers agree?. Arch Intern Med.

[25] Malhotra C, Farooqui MA, Kanesvaran R (2015). Comparison of preferences for end-of-life care among patients with advanced cancer and their caregivers: a discrete choice experiment. Palliat Med.

[26] Gottesman LE, Bourestom NC (1974). Why nursing homes do what they do. Gerontologist.

[27] Sahadevan S, Tan NJL, Chan SP (2000). Diagnostic performance of two mental status tests in the older Chinese: influence of education and age on cut-off values. Int J Geriatric Psychiatry.

[28] Kuhfeld WF, Tobias RD, Garratt M. Efficient experimental design with marketing research applications. J Mark Res. 1994:545–57.

[29] Özdemir S, Mohamed AF, Johnson FR (2010). Who pays attention in stated-choice surveys?. Health Econ.

[30] McIntosh E, Ryan M (2002). Using discrete choice experiments to derive welfare estimates for the provision of elective surgery: implications of discontinuous preferences. J Econ Psychol.

[31] Luo N, Wang P, Thumboo J (2014). Valuation of EQ-5D-3L health states in Singapore: modeling of time trade-off values for 80 empirically observed health states. Pharmacoeconomics.

[32] Tajima R, Kondo M, Kai H (2010). Measurement of health-related quality of life in patients with chronic kidney disease in Japan with EuroQol (EQ-5D). Clin Exp Nephrol.

[33] Van Biesen W, van der Veer SN, Murphey M (2014). Patients’ perceptions of information and education for renal replacement therapy: an independent survey by the European kidney Patients' Federation on information and support on renal replacement therapy. PLoS One.

[34] Ladin K, Lin N, Hahn E, et al. Engagement in decision-making and patient satisfaction: a qualitative study of older patients' perceptions of dialysis initiation and modality decisions. Nephrol Dial Transplant. 2017;32(8):1394–401. 10.1093/ndt/gfw307 PMC5837335 27576590

