# Supplementary material for: Family dynamics in a multi-ethnic Asian society: comparison of elderly CKD patients and their family caregivers experience with medical decision making for managing end stage kidney disease
Source: BMC Nephrol. 2019 Mar 1;20:73. doi: 10.1186/s12882-019-1259-4 (PMC6397496; doi:10.1186/s12882-019-1259-4)
Supplement: Supplementary file 1 — Patient Survey Instrument. The survey instrument administered to the patient participants in this study. (PDF 273 kb) [file 12882_2019_1259_MOESM1_ESM.pdf]

Supplementary Material: Patient Survey Instrument

Quantifying Treatment Preferences of Elderly Chronic Kidney Disease Patients and their Family Caregivers
Patient Questionnaire

For interviewer: Fill in the spaces that you can at the beginning of the interview, then ente Time Ended and Total Interview Time after completing the survey.

PARTICIPANT CODE: (FROM CONSENT FORM) [ ] DATE OF SURVEY (DD/MM/YYYY): _____/_____/________

TIME STARTED: ________ TIME ENDED: ________ TOTAL INTERVIEW TIME: ______________ MIN

INTERVIEWER NAME: __________________________

SURVEY CONDUCTED IN: ☐1 ENGLISH ☐2 MANDARIN ☐3 MALAY ☐4 TAMIL

INTRODUCTION

We are conducting a survey to understand how patients and their family caregivers think about treatments for kidney failure. Even if you do not have kidney failure, we would like to know your opinions as a patient with chronic kidney disease. Your opinions are important to the success of this study. The survey usually takes about 30 minutes.

There are no right or wrong answers and you do not have to answer any questions that make you feel uncomfortable. Information about who you are and your answers will be kept confidential. Only group data will be reported.

INFORMATION FROM MEDICAL RECORDS

Interviewer: Please record the participant's information from patient's medical record file

GFR (Glomerular Filtration Rate) : ☐ **ml/min**

Age as of last birthday: ☐

[Interviewer: Please end interview if age is less than 65 years and GFR > 59.

SECTION S: SCREENING QUESTIONS

S1 **Have you ever been diagnosed with chronic kidney disease?**

☐ 1 Yes ☐2 No ☐3 Not sure

[Interviewer: Please end interview if the answer is not "Yes"]

S2 **Are you currently on dialysis?**

☐ 1 Yes ☐2 No

[Interviewer: Please end interview if participant answers "Yes"]

Abbreviated Mental Test (AMT)

READ OUT: Before starting the main questionnaire, we would just like to ask you a few questions. These questions are about memory. The questions may seem unusual, but they are routine questions we ask of everyone. Some of the questions are very easy and some are difficult, so do not be surprised if you have trouble with some of them.

Please try to answer these questions correctly and to the best of your knowledge.

Have you attained more than six years of school education?

1 Yes 2 No

Please remember the following phrase: "37 Bukit Timah Road." I will be asking you to repeat the phrase to me later.

Questions	Response	Correct only when:
a. **What is the present year**	Correct / Incorrect	**Western calendar , i.e. 20__**
b. **What time is it now?**	Correct / Incorrect	**Within 1 hour**
c. **What is your age?**	Correct / Incorrect	**For Chinese, +1 yr is usually the norm and hence acceptable. Refer to patient list.**
d. **What is your date of birth?**	Correct / Incorrect	**Western year +/- month and day, refer to the SAC list. Refer to patient list**
e. **What is your home address?**	Correct / Incorrect	**Complete address excluding postal code, refer to patient list**
f. **Where are we now?**	Correct / Incorrect	**For community survey, "my home" or "my son's home" etc. is probably acceptable.**
g. **Who is Singapore's present Prime Minister?**	Correct / Incorrect	**Lee Hsien Loong**
h. **Show 'Showcard 1'. What is her occupation?**	Correct / Incorrect	**Nurse or doctor ok**
i. **Now, count backwards from 20 to 1**	Correct / Incorrect	
j. **Please recall memory phrase**	Correct / Incorrect	

<u>Interviewer:</u> Please total up the number of questions correct. Then see the table below to determine if the respondent has passed the cognitive health section based on his or her age and education status. If they have too many questions wrong, please terminate the interview.

Questions correct: ___________________

Number of questions needed correct to continue to questionnaire:

	**60-74 Years Old**	**75+ years Old**

**0 to 6 years of education**	8 or more correct	6 or more correct
**More than 6 years of education**	9 or more correct	9 or more correct

[Interviewer: Please end interview if participant fails AMT]

SECTION A: BACKGROUND QUESTIONS

A1 Gender

☐1 Male ☐2 Female

A2 Which ethnic group do you belong to?

☐1 Chinese
☐3 Indian
☐2 Malay
☐4 Other (Please specify:_______________)

A3 What is your current marital status?

☐1 Married
☐4 Divorced
☐2 Separated
☐5 Never married
☐3 Widowed

A4 What is your highest education level completed?

☐1 No formal education
☐5 Junior college/ Polytechnic/ Diploma
☐2 Primary
☐6 University and above
☐3 Secondary
☐7 Don’t know/ Can’t remember
☐4 Vocational/ ITE

A5 **Have you ever been diagnosed with any of the following illnesses/health conditions? Please check all that apply.**

Illness/Conditions	Yes	No	Not Sure
1. **Diabetes**	☐	☐	☐
2. **Cerebrovascular Disease such as stroke**	☐	☐	☐
3. **Heart Failure**	☐	☐	☐
4. **Heart attack, heart blood vessel narrowed or blocked**	☐	☐	☐
5. **Irregular heartbeat, also called dysrhythmia**	☐	☐	☐
6. **Other heart conditions, such as heart valve problems**	☐	☐	☐
7. **Chronic Obstructive Pulmonary Disease such as emphysema or chronic bronchitis**	☐	☐	☐
8. **Liver Disease**	☐	☐	☐
9. **Gastrointestinal Bleeding**	☐	☐	☐
10. **Poor circulation or blocked blood vessels**	☐	☐	☐
11. **Cancer**	☐	☐	☐

SECTION B: KIDNEY FAILURE TREATMENT OPTIONS

Dialysis

Healthy kidneys remove harmful waste from the blood. Without kidneys, the waste would build up and people would get sicker and eventually die. When kidneys start failing, people may choose to go on dialysis. Dialysis uses a machine to remove some of the waste from the blood so that people can do more of their daily activities and hopefully stay healthy longer.

There are two kinds of dialysis. For blood dialysis (also called haemodialysis), patients need to go to a hospital or a dialysis centre. Water-bag dialysis (also called peritoneal dialysis) can be done at home. Patients on either type of dialysis also have to make changes to their diet, drink less liquid and take medicines.

B1 **Have you heard of water-bag dialysis?**

☐1 Yes ☐2 No ☐3 Not sure

B2 **Have you heard of blood dialysis?**

☐1 Yes ☐2 No ☐3 Not sure

Non-dialysis management

Non-dialysis management is also an option for kidney failure. Under non-dialysis management, the patient uses diet and medicines without dialysis. It does not remove waste from the blood as dialysis does. Instead, it tries to control symptoms and maintain quality of life while the disease runs its natural course.

B3 **Did you know that non-dialysis management is also an option for patients with kidney failure?**

☐1 Yes ☐2 No ☐3 Not sure

B4 **Have you participated in a counselling session with a Renal Coordinator given at the kidney clinic?**

☐1 Yes ☐2 No ☐3 Not sure

SECTION C: INTRODUCTION TO TREATMENT FEATURES

In this section we will ask you to think about some features of treatments for kidney failure. Even if you do not have kidney failure, please assume that you have kidney failure when answering the following questions. The treatment features are:

- *Type of treatment*
- *Expected survival with this treatment*
- *Quality of daily life*
- *Expected out-of-pocket health care cost per month*

Type of treatment

Later in this survey, we will ask you to think about three types of treatment for kidney failure:

- **In-centre blood dialysis**:
  - You would need to go to a dialysis centre 3 times a week and each dialysis treatment will take 4 hours.
  - You would receive 2 needle sticks per session (6 needle sticks per week) which can be painful.
  - The risk of infection from receiving blood dialysis is very small.
- **Water-bag dialysis at home**:
  - You would need to have dialysis every day at home. You can do water-bag dialysis either 4 times throughout the day or for 8 hours at night while you sleep.
  - You would have no pain during water-bag dialysis.
  - The risk of infection from receiving water-bag dialysis is very small but may be higher for patients with hygiene problems.
- **Non-dialysis management only**:
  - You would not be using dialysis. Instead, you would take food supplements and medicines, and limit liquids to control your symptoms.
  - The symptoms will get worse over time.

C1 **If you were to start *blood dialysis* in the next couple months, what is the most likely way you would travel to a dialysis centre?**

☐1 Bus and/or MRT

☐2 Taxi

☐3 Private car

☐4 Walking without using any other transportation

☐5 Others, please specify: ________________

**C2**	**If you were to start *blood dialysis* in the next couple months, how often would someone be able to help you to go to the dialysis centre?**

☐1 All the time

☐2 Most of the time

☐3 Some of the time

☐4 Never

**C3**	**[SKIP IF C2 = 4] Who would be most likely to take you to the dialysis centre?**

☐1	Husband or wife	☐5	Maid
☐2	Son or daughter	☐6	Friend
☐3	Son-in-law or daughter-in-law	☐7	Nurse or other health worker
☐4	Other family member	☐8	Others, specify: ________________

**C4**	**If you were to start *water-bag dialysis* in the next couple months, how often would someone be able to help you to have dialysis at home?**

☐1 All the time

☐2 Most of the time

☐3 Some of the time

☐4 Never

**C5**	**[SKIP IF C4 = 4] Who would be most likely to help you with *water-bag dialysis* at home?**

☐1	Husband or wife	☐5	Maid
☐2	Son or daughter	☐6	Friend
☐3	Son-in-law or daughter-in-law	☐7	Nurse or other health worker
☐4	Other family member	☐8	Others, specify: ________________

Expected survival

Later in the survey, we will ask you to think about how long older adults live with kidney failure. Now we would like to get your opinion on an imaginary patient's survival under different circumstances.

Mr/Ms Law is _____ years old and has kidney failure. He/She has similar other health problems as you do. He/She can choose either dialysis or non-dialysis management to treat the symptoms from kidney failure.

C6 **What is your best guess as to how many more years someone like Mr/Ms Law will live using…**

i. Dialysis?

_______ years ☐ Not sure/ Don't know

ii. Non-dialysis management only (no switching to dialysis)?

_______ years ☐ Not sure/ Don't know

C7 **[ASK IF ANY C6 = Don't know] Under which treatment do you think Mr/Ms Law would have a longer life?**

☐1 Dialysis

☐2 Non-dialysis management only

☐3 Not sure/ Don't know

C8 **Until what age do you think a person who is your age and gender <u>without chronic kidney disease</u> is likely to live (please provide your best guess)?**

☐ **years old**

☐ Not sure/ Don't know

Quality of daily life
Later in the survey, we will ask you to think about the quality of daily life using the following definitions:

- **Poor**: It is not possible for you to do activities that are important to you.
- **Fair**: You can do some activities that are important to you.
- **Good**: You can do most activities that are important to you.
- **Very Good**: You can do all of the activities that are important to you.

C9 <u>Over the past month</u>, how would you rate your quality of daily life based on the definitions above?

☐1 Poor
☐2 Fair
☐3 Good
☐4 Very good

C10 What do you think your quality of daily life would be like if you had kidney disease and you were using …

i. Blood dialysis?

☐1 Poor
☐2 Fair
☐3 Good
☐4 Very good
☐5 Not sure/ Don't know

ii. Water-bag dialysis?

☐1 Poor
☐2 Fair
☐3 Good
☐4 Very good
☐5 Not sure/ Don't know

iii. Non-dialysis management only?

☐1 Poor
☐2 Fair
☐3 Good
☐4 Very good
☐5 Not sure/ Don't know

C11 [ASK IF ANY C10 = Don't know] Which treatment do you think offers the best quality of daily life?

☐1 Water-bag dialysis at home
☐2 In-centre blood dialysis
☐3 Any form of dialysis
☐4 Non-dialysis management only
☐5 They are all about the same
☐6 Don't know

Expected out-of-pocket medical cost per month
Out-of pocket costs refer to the *total amount you or your family have to pay for all medical costs* after

- Deductions from MediSave account (personal or family)
- Claims from MediShield and any other health insurance
- Other subsidies (e.g. Medifund, National Kidney Foundation)

Later in the survey, we will ask you to think about **out-of-pocket costs in a typical month** for **all your health problems**, including kidney failure.

C12 We would like you to indicate what you think your out-of-pocket costs for all your health problems will be in a typical month if you had kidney failure and if you were using….

i. Blood dialysis?

$________ ☐ Not sure/ Don't know

ii. Water-bag dialysis?

$________ ☐ Not sure/ Don't know

iiii. Non-dialysis management only?

$________ ☐ Not sure/ Don't know

C13 [ASK IF ANY C12 = Don't know] Under which treatment do you think you will have the highest out-of-pocket cost for all your health problems in a typical month?

☐1 Water-bag dialysis at home
☐2 In-centre blood dialysis
☐3 Non-dialysis management only
☐4 Don't know

Doctor's Recommendation
Doctors may recommend a particular treatment for kidney failure. The recommendation might or might not be based on the same concerns you and your family have about your health and health care.

C14 When making decisions about your health and health care, *who usually makes the final decisions*?

☐1 Me only	☐5 Me and my doctor only
☐2 My family only	☐6 My family and my doctor only
☐3 My doctor only	☐7 Me, my family and my doctor
☐4 Me and my family only	☐8 Other. Please specify:__________________

C15 When making decisions about your health and health care, *who do you want* to make the final decisions?

☐1 Me only	☐5 Me and my doctor only
☐2 My family only	☐6 My family and my doctor only
☐3 My doctor only	☐7 Me, my family and my doctor
☐4 Me and my family only	☐8 Other. Please specify:__________________

C16 If there is a disagreement about the care of chronic kidney disease, *whose opinion do you think will be most important* in deciding what to do?

☐1 My own
☐2 My family's
☐3 My doctor's

SECTION D: WHICH SCENARIO WILL YOU CHOOSE?

In the next 9 questions, we will show you two possible treatments for kidney failure. Even if you do not have kidney failure, please assume that you have kidney failure when answering these questions. Please tell us which treatment you would choose if these were the only options available for you. Assume that once you make a decision it is final; you cannot change your mind later.

Please think carefully about your choice. Your answers will help increase our understanding of trade-offs that patients make when faced with decisions about treatment for kidney failure.

Please think about whether you and your family can afford paying the cost of the treatment before making your choice.

There are no right or wrong answers.

EXAMPLE QUESTION

If these were the only treatment options, which one would you choose?

	Profile A	Profile B
**Type of treatment**	In-centre blood dialysis<br><br>(3 times a week)	Non-dialysis management
**Expected survival**	6 years	3 years
**Quality of daily life**	Fair	Fair
**Expected out-of pocket cost per month**	S$ 2,500 per month	S$ 450 per month

If you choose **Treatment A**, you would have dialysis at a centre 3 times a week, and each dialysis treatment will take 4 hours. The expected survival would be 6 years and you would have a fair quality of daily life. Expected costs would be S$2,500 per month for all your health care costs.

If you choose **Treatment B**, you would receive supplements and medication to manage your symptoms. The expected survival would be 3 years and you would have a fair quality of daily life. Expected costs would be S$450 per month for all your health care costs.

You do not need to provide an answer for this question. This is just an example.

D1. If these were the only treatment options, which one would you choose?

	Profile A	Profile B
**Type of treatment**	In-centre blood dialysis<br><br>(3 times a week)	Non-dialysis management
**Expected survival**	3 years	3 years
**Quality of daily life**	Good	Good
**Expected out-of pocket cost per month**	$1,500	$1,000
**If these were the only options, which would you choose?**	O	O

D2. If these were the only treatment options, which one would you choose?

	Profile A	Profile B
**Type of treatment**	In-centre blood dialysis<br><br>(3 times a week)	Non-dialysis management
**Expected survival**	10 years	1 year
**Quality of daily life**	Very good	Very good
**Expected out-of pocket cost per month**	$700	$500
**If these were the only options, which would you choose?**	O	O

D3. If these were the only treatment options, which one would you choose?

	Profile A	Profile B
**Type of treatment**	Water bag dialysis at home<br><br>(Every day or night)	Non-dialysis management
**Expected survival**	3 years	1 year
**Quality of daily life**	Poor	Good
**Expected out-of pocket cost per month**	$3,000	$250
**If these were the only options, which would you choose?**	O	O

D4. If these were the only treatment options, which one would you choose?

	Profile A	Profile B
**Type of treatment**	In-centre blood dialysis<br><br>(3 times a week)	Water bag dialysis at home<br><br>(Every day or night)
**Expected survival**	1 years	10 years
**Quality of daily life**	Very good	Poor
**Expected out-of pocket cost per month**	$1,500	$700
**If these were the only options, which would you choose?**	O	O

D5. If these were the only treatment options, which one would you choose?

	Profile A	Profile B
**Type of treatment**	Water bag dialysis at home<br><br>(Every day or night)	Non-dialysis management
**Expected survival**	6 years	1 year
**Quality of daily life**	Poor	Fair
**Expected out-of pocket cost per month**	$7,000	$500
**If these were the only options, which would you choose?**	O	O

D6. If these were the only treatment options, which one would you choose?

	**Profile A**	**Profile B**
**Type of treatment**	Water bag dialysis at home<br><br>(Every day or night)	Non-dialysis management
**Expected survival**	6 years	3 years
**Quality of daily life**	Fair	Poor
**Expected out-of pocket cost per month**	$3,000	$2,000
**If these were the only options, which would you choose?**	O	O

D7. If these were the only treatment options, which one would you choose?

	Profile A	Profile B
**Type of treatment**	In-centre blood dialysis<br><br>(3 times a week)	Non-dialysis management
**Expected survival**	1 year	3 years
**Quality of daily life**	Very good	Good
**Expected out-of pocket cost per month**	$1,500	$1,000
**If these were the only options, which would you choose?**	O	O

D8. If these were the only treatment options, which one would you choose?

	Profile A	Profile B
**Type of treatment**	Water bag dialysis at home<br><br>(Every day or night)	Non-dialysis management
**Expected survival**	10 years	3 years
**Quality of daily life**	Poor	Good
**Expected out-of pocket cost per month**	$700	$1,000
**If these were the only options, which would you choose?**	O	O

D9. If these were the only treatment options, which one would you choose?

	Profile A	Profile B
**Type of treatment**	In-centre blood dialysis<br><br>(3 times a week)	Non-dialysis management
**Expected survival**	3 years	1 year
**Quality of daily life**	Fair	Fair
**Expected out-of pocket cost per month**	$3,000	$250
**If these were the only options, which would you choose?**	O	O

D10 If your doctor strongly recommended ____ and if you could afford this treatment, would you take the doctor recommended treatment?

☐1 Definitely would

☐2 Probably would

☐3 Probably would not

☐4 Definitely would not

SECTION E: CURRENT HEALTH STATUS

E1 By placing a tick in one box [✓] in each group below, please indicate which statements best describe your own health state <u>today</u>.

i. Mobility

- ☐ 1 I have no problems in walking about
- ☐ 2 I have some problems in walking about
- ☐ 3 I am confined to bed

ii. Self-Care

- ☐ 1 I have no problems with self-care
- ☐ 2 I have some problems washing or dressing myself
- ☐ 3 I am unable to wash or dress myself

iii. Usual Activities *(e.g. work, study, housework, family or leisure activities)*

- ☐ 1 I have no problems with performing my usual activities
- ☐ 2 I have some problems with performing my usual activities
- ☐ 3 I am unable to perform my usual activities

iv. Pain/Discomfort

- ☐ 1 I have no pain or discomfort
- ☐ 2 I have moderate pain or discomfort
- ☐ 3 I have extreme pain or discomfort

v. Anxiety/Depression

- ☐ 1 I am not anxious or depressed
- ☐ 2 I am moderately anxious or depressed
- ☐ 3 I am extremely anxious or depressed

E2 To help people say how good or bad a health state is, we have drawn a scale (rather like a thermometer) on which the best state you can imagine is marked 100 and the worst state you can imagine is marked 0.

We would like you to indicate on this scale how good or bad your own health is today, in your opinion. Please do this by drawing a line from the BLACK BOX below to whichever point on the scale indicates how good or bad your health state is today.

Your own health state today

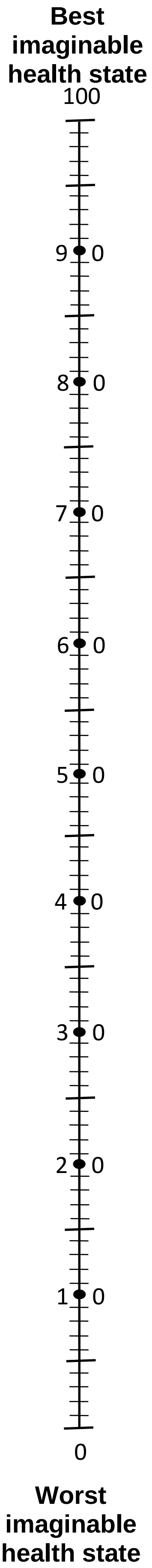

SECTION F: ADDITIONAL BACKGROUND QUESTIONS

F1 What is your CURRENT employment situation:

☐1 Paid full-time
☐2 Paid part-time
☐3 Self-employed
☐4 Homemaker
☐5 Volunteer
☐6 Retired due to health problems
☐7 Retired – non-health reasons
☐8 Unemployed, looking for work
☐9 Unemployed, not looking for work

F2 What type of housing are you living in?

☐1 HDB/ JTC flat (1-2 room)
☐2 HDB/ JTC flat (3 room)
☐3 HDB/ JTC flat (4 room)
☐4 HDB/ JTC flat (5 room and above/ HUDC/ Executive)
☐5 Condominium/ Private flat
☐6 Bungalow/ semi-detached/ terrace house
☐7 Shophouse
☐8 Others, specify____________________

F3 How many persons live in your household?
[*Interviewer:* *This includes all who live in the household presently, as well as those who have this address recorded in their NRIC, excluding tenants. e.g. including yourself, maid and those who are temporarily hospitalised, children who are living in a hostel within Singapore, and household members who are currently in National Service/ Reservist training, etc.*]

[ ] **Number of persons**

F4 How many persons in your household earn income?
[*Interviewer:* *Exclude domestic helpers.*]

[ ] **Number of persons**

F5 What is your religion?

☐1 Christian
☐2 Buddhist
☐3 Taoist
☐4 Muslim
☐5 Hindu
☐6 Sikh
☐7 Other, specify:__________________
☐8 No religion
☐9 Free thinker

**F6**	**[SKIP IF F5 = 8 or 9] At a time of suffering and distress, to what extent do you find strength and comfort from your religion?**<br>☐1 None ☐3 Some<br>☐2 A little ☐4 A great deal

SECTION G: BEQUEST MOTIVES

**GI.**	**How important is it for you to leave money and/or property to your family or to charity once you pass on?**<br>☐1 Essential, I cannot imagine not leaving anything after I am gone<br>☐2 Highly desirable, I will try my best and will be disappointed if I'm unable to.<br>☐3 Desirable, I will try but will not be disappointed if I'm unable to.<br>☐4 Not important at all, it does not matter to me.

SECTION H: PERSONAL VALUES AND BELIEFS

**H1**	Please state how strongly you agree or disagree with the following statements:
**i.**	**I prefer traditional medicine instead of western medicine.**<br>☐1 Strongly disagree ☐2 Disagree ☐3 Not sure ☐4 Agree ☐5 Strongly agree
**ii.**	**I cannot change my fate so there is no point to have dialysis.**<br>☐1 Strongly disagree ☐2 Disagree ☐3 Not sure ☐4 Agree ☐5 Strongly agree
**iii.**	**If I do not have dialysis in this life, I may suffer the same problem in the next life.**<br>☐1 Strongly disagree ☐2 Disagree ☐3 Not sure ☐4 Agree ☐5 Strongly agree
**iv.**	**I fear that I will be a burden to my family if I have dialysis regularly.**<br>☐1 Strongly disagree ☐2 Disagree ☐3 Not sure ☐4 Agree ☐5 Strongly agree
**v.**	**It is possible for me to be healthy without dialysis.**<br>☐1 Strongly disagree ☐2 Disagree ☐3 Not sure ☐4 Agree ☐5 Strongly agree
